# Complete genome of a *Corynebacterium ulcerans* clinical isolate with two novel toxin gene alleles isolated from a leg ulcer

**DOI:** 10.1128/mra.00857-25

**Published:** 2025-12-17

**Authors:** Anne-Marie Bernier, Mark Unger, Tamara Burdz, Jennifer R. Tanner

**Affiliations:** 1 National Microbiology Laboratory-CSCHAH, Public Health Agency of Canada, Winnipeg, Manitoba, Canada; 2 Department of Biology, Université de Saint-Boniface 33529, Winnipeg, Manitoba, Canada; University of Pittsburgh School of Medicine, Pittsburgh, Pennsylvania, USA

**Keywords:** *Corynebacterium*, toxin, complete genome, cutaneous infection

## Abstract

We report on the complete genome sequence of a clinical isolate of *Corynebacterium ulcerans* bearing two different and novel toxin gene alleles.

## ANNOUNCEMENT

Only two reports exist of *Corynebacterium ulcerans* strains harboring two diphtheria toxin genes (1, 2). The Special Bacteriology Unit at the National Microbiology Laboratory in Canada received a pure culture of *C. ulcerans* (NML 200269) from a leg ulcer from a human patient in Ontario, Canada for diphtheria toxin testing. Identification via DNA-directed RNA polymerase subunit beta (rpoB) gene sequencing confirmed the species. The isolate was toxin gene-positive by qPCR (3) and toxigenic per modified Elek test (4).

The culture was stored at −80°C in Microbank tubes (Pro-Lab Diagnostic) and grown on Columbia blood agar base with 5% sheep blood at 35°C in 5% CO_2_ for 24 h. Unless noted, default parameters for all methods were used. DNA was extracted using the Qiagen QIAmp DNA Mini Kit, and Illumina paired-end (PE) libraries were prepared using an in-house validated and optimized reduced volume NexteraXT Library Prep Kit protocol based on Hackflex principles (5). Sequencing was done on a NextSeq2000 (600 cycles, 2 × 300 bp). Read quality was assessed with FastQC (v.0.72)(https://www.bioinformatics.babraham.ac.uk/projects/fastqc/), and reads were filtered for phred quality >=Q15 using FastP (v.0.23.2) (6) (Table 1). Assembly of Illumina reads was performed using Shovill (v.1.1.0) (https://bio.tools/shovill) with Spades as the assembler (v.1.1.0) (7) (Table 1). Mapping of Illumina PE reads to the toxin gene, which assembled into one small orphan contig, revealed hybrid SNPs suggesting the presence of two different toxin genes that could not be assembled individually using Illumina technology alone (2).

**TABLE 1:** Sequencing data and relevant features of *Corynebacterium ulcerans* strain NML200269 (Pasteur ID 7247)

MLST	ST 1096 (novel allele leuA 125)
cgMLST ulcerans	cgST 845
# bp total	2,536,927
# replicons	1
GC content (%)	53.3
NCBI bioproject	PRJNA1255620
NCBI Ref Seq Assembly	GCF_050743115.1
NCBI genbank accession	NZ_CP189778.1
Genome coverage (hybrid assembly)	740×
Illumina sequencing depth	255×
Toxin gene 1 (XUQ17417.1)	start 205098bp—novel allele 42
Toxin gene 2 (XUQ17424.1)	start 216518bp—novel allele 41
BLASTN *tox* 1 vs *tox* 2	1,669/1,683 matches and 0 gaps: 99.1% ID
BLASTP Tox one vs Tox 2	557/560 matches and 0 gaps: 99.46% ID
# MinION reads	234,468
# MinION bases	1,357,541,522
MinION read length N50	9447
# Illumina reads	2,182,244
# Illumina bases	557,526,010
SRA accessions MinION	SRR33319770
SRA accessions Illumina	SRR33319771
PGAP annotation:	
# Genes	2,303
# CDS (with protein)	2,238
# Complete rRNAs	12
# tRNA	50
# CRISPR arrays	2
# ncRNA	3
# complete phage	one complete phage: position 171028bp-194151bp

To resolve this, we applied long read sequencing using Oxford Nanopore Technology (ONT). DNA was extracted as described above, and 400 ng (Qubit fluorometry) of unsheared, non-size-selected gDNA was used for the library preparation using the ONT Ligation Sequencing gDNA Native Barcoding Kit 24 V14 (SQK-NBD114.24). The FFPE DNA repair step was omitted, and AMPure cleanup elutions were performed at 37°C with mixing at 350 rpm. Sequencing was performed using an ONT MinION Mk1C with a R10 FLO-MIN114 flow cell. Base calling, adaptor trimming, and demultiplexing were performed with Dorado (v. 7.6.7) using the super high-accuracy model without error correction (https://github.com/nanoporetech/dorado). Read quality was assessed using Nanoplot (v1.28.2) (8) (Table 1), and reads were filtered to a minimum length of 1,000 bp using Nanoq (0.10.0) (9).

A hybrid assembly was produced in Unicycler (Galaxy v. 0.4.8.0) (10) from filtered Nanopore and Illumina PE fastq reads using normal bridging mode. In Unicycler, the genome was rotated to start at the *dnaA* gene, and pilon (v.1.20.1) (11) was used for polishing. The assembly graph visualized in Bandage (v.0.8.1) (12) confirmed the genome to be circular. Assembly quality was assessed with Quast (v.5.0.2) (13). The isolate was assigned the novel MLST ST 1096 (14) and the novel cgMLST_ulcerans cgST 845 (https://bigsdb.pasteur.fr/diphtheria/) and confirmed to be *C. ulcerans* via MLST (15).

**Fig 1:**
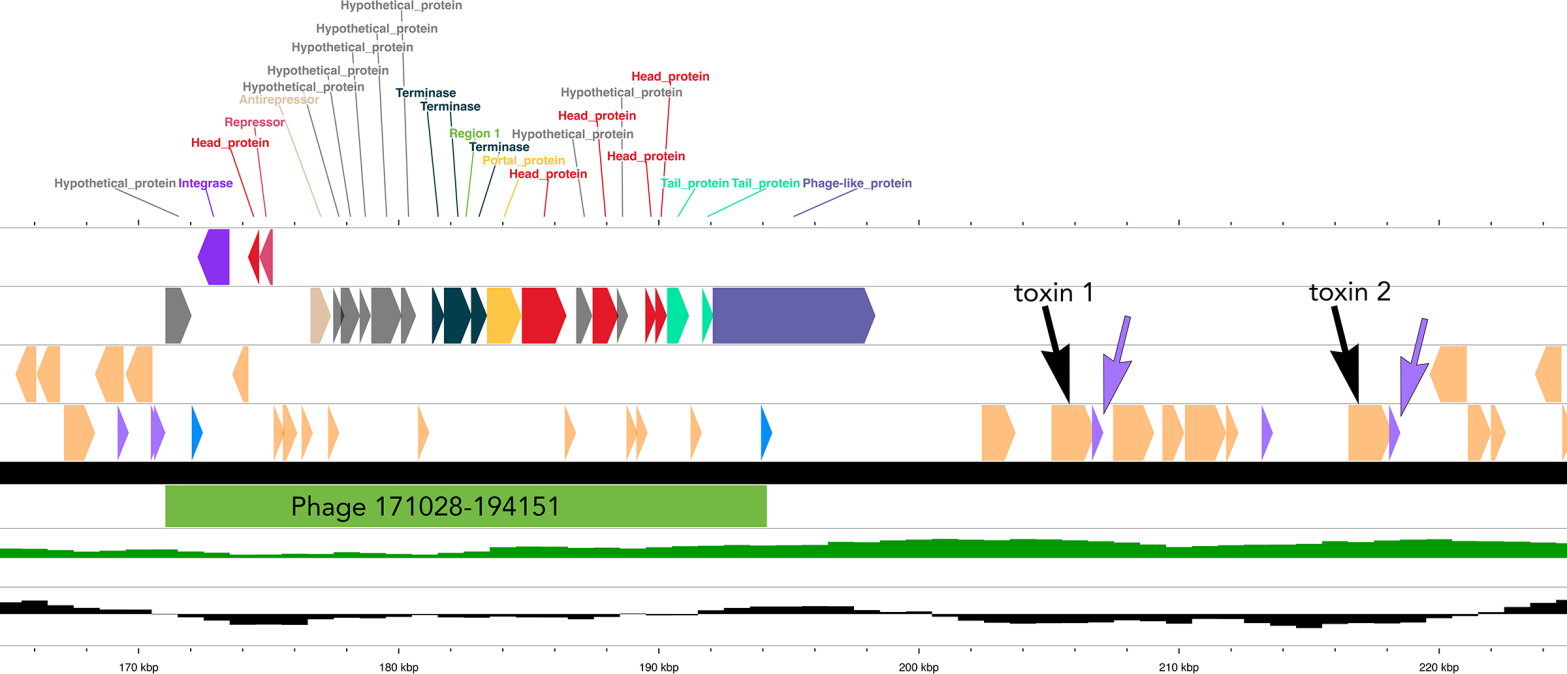
Region 165 to 225 kbp of the NML200269 genome annotated and visualized with Phastest (17) including the phage at position 171028bp-194151bp (in green), the two toxin genes are identified (black arrows, starting at 205098bp and 216518bp) and their associated pseudo-tRNA (purple arrows and triangles). Peach-colored shapes are annotated bacterial genes; the blue triangles are the phage attachment sites; and the purple triangles are tRNAs.

Two toxin genes were identified by PGAP annotation (16) (Table 1), and PHASTEST (17) mapped each gene adjacent to an identical Pseudo-tRNA just downstream of the single prophage (Fig. 1). These novel toxin alleles, 41 and 42 (https://bigsdb.pasteur.fr/diphtheria/), share 99.1% identity by BLASTN and 99.5% identity by BLASTP for toxin protein sequences (Table 1). This is the first report of a *C. ulcerans* strain harboring two different toxin gene alleles.

## Data Availability

The whole genome sequence of NML200269 is available with NCBI GenBank bioproject PRJNA1255620, and NCBI GenBank accession numbers are in Table 1.
